# The Treatment of Yellow Fever

**Published:** 1888-10

**Authors:** 


### THE TREATMENT OF YELLOW FEVER.

As the subject of yellow fever is being much discussed at present, a few words by one whose fortune or misfortune, it has been to see a good deal of the disease, may not be out of place, or uninteresting.

Of all the proposed "plans" of treatment which have recently appeared in the medical press, that of the venerable and talented Dr. R. H. Day, of Baton Rouge, La., strikes us as being the most rational, and his views coincide very nearly with our own,

In yellow fever there is no "royal road to success" in treatment: the individuality of each case must be studied. Our experience leads us to conclude that there are certain cases occurring in all epidemics, which *are necessarily fatal;* and that the average mortality, under any plan of treatment, will never be materially lessened.

The success of treatment depends largely on the stage of the disease at which the physician sees the case, but mostly, on the *quantity* of the poison acquired by the patient, relative to his powers of resistance. We daily inhale, and otherwise introduce into our blood, toxic elements, or it is being generated, or liberated, within the system, in the ordinary physiological processes, *e. g.* urea, in the secretion and excretion of urine, and carbonic di-oxide in respiration; and we are as constantly exhaling or eliminating said poisons; and thus the equilibrium, called "health," is maintained. Every person's powers of eliminating poison from the system are not equal; his resistance to the invasion of disease varying according to age, sex, temperament, state of body and mind, etc. We all know the depressing influence of fear; it predisposes, or lays one liable to an attack during an epidemic,—his powers of resistance or ability to "throw off" being weakened by the depressed state of the nervous system. Anything that depresses or shocks the nervous system, will have this effect. Witness the occurrence of malarial fever after childbirth, in a person who lives constantly in malarial atmosphere, yet who keeps up a balance, by elimination, as fast as introduced; when no longer able to rid the system of the poison, an "explosion" takes place, as it were, and the poison "gets in its work," Were every person's resistant and eliminant powers equal, it would be possible, perhaps, to formulate some rule of practice of universal application; but, as it is not, the severity of an attack of yellow fever, for instance, will depend upon the quantity of the poison acquired, in referrence to the individual's stamina; the larger the dose, (*ceteris paribus*), the severer the attack. What would produce only a mild fever, of short duration, in one person, might prove fatal in another, and in persons of weak vital powers, an overwhelming "dose" must be necessarily fatal. Like any other poison, a small quantity might be overcome by the *vis medicatrix naturæ;* a large quantity may necessitate the assistance of eliminating or stimulating drugs or measures, and a still larger quantity defies both, system and doctor; it overwhelms the nervous system, and renders all treatment nugatory. This is seen in opium narcosis; where the quantity is immense, in proportion to the stamina of the patient, and the case seen late; no power on earth can arouse the nervous system; it is overwhelmed, paralysed, and death is inevitable. So with yellow fever; the result depends on the quantity of the poison acquired, (other things being equal.) Some cases, where the quantity of poison is small, and the system vigorous, recover readily, without assistance; others produce grave pathological changes, which are difficult to meet, and where the doctor must "assist nature"; and others are doomed, from the inception.

There are cases also in which complications arise, and structural changes occur, the result of which would be fatal, independent of the fever poison; the congestion of the kidneys and the choking up of the uriniferous tubules, for instance,—whereby the patient falls a victim of retained urea, and dies, not of the yellow fever poison, *per se*, but of uric toxæmia. A skilled and experienced physician will recognize a condition that will inevitably lead to suppression, and will, by proper remedies, prevent it. He knows that the intense lumbar pains, which occur often, mean congestion of the kidneys; they are crying aloud for relief, and if not relieved, the uriniferous tubes become blocked with plastic matter, or fat globules, which, with the congestion, constitute a mechanical obstruction, and the further secretion of urine becomes a *physical impossibility*, and death from uræmic poison becomes inevitable. How long would a person live, even if in the best of health, were it possible to ligate the ureters? The effect is practically the same; the excretion and escape of the urine, and other poisonous matter, is cut off. This is the only point where we differ with Dr. Day. He speaks of the relief of suppression. One had as well hope to get water from a dried bladder, as to force the excretion of urine ater forty-eight hours of suppression. In an extensive experience in the epidemic of '67 in Galveston, and that of '78 in Lake Village, Miss., and elsewhere, we never saw a case of suppression relieved after forty-eight hours.

Dr. Day clearly recognizes a fact which we have always insisted upon, to-wit: that there are several kinds of yellow fever, so to speak: that is, the poison having invaded the system, and produced fever, seems to have an elective affinity for one of the three organs,—brain, stomach or kidneys; and according as the stress falls on one or the other, will the symptoms differ, and furnish indications for treatment; and different, also, will be the mode of fatal termination, in case of death. If the brain be selected, the symptoms will be those of *congestio cerebri*, (and perhaps, later, inflammation of the brain,) and we too, have "seen cases saved by opening the temporal artery," as Dr. Day says. If on the stomach, we will have a case of specific gastritis, (preceded, of course, by congestion), and these are the cases that have black vomit, as a rule; if on the kidneys, the symptoms point to congestion of these organs, and, from the first, there is a tendency to suppression. Here is where death from uraemic coma is to be apprehended, and the suppression is to be prevented, as it cannot be relieved. Would any rational man open the temporal artery in a case of this kind? or in gastritis? Hence, we say, there is, and can be, no routine treatment applicable to all cases. The doctor must exercise common sense, diagnose the *kind* of yellow fever, (which is perfectly plain,) estimate the probable amount of poison at work, by the symptoms, take into consideration the stamina, or resistant powers of the individual, and adapt his treatment to the case.

Unlike other fevers, the danger period is when the fever goes off. The patient will feel well, and will insist on getting up; he thinks it "will do him good to stretch his legs," and he will almost invariably take advantage of absence or inattention of attendants to do it. Just as surely will he pay the penalty with his life. Absolute quiet is the first essential upon the decline of the fever; and mild stimulants, in small quantities,—cool champagne, not iced, being the best, in our hands.

Medicines do little good; the *cure* for yellow fever does not exist. We believe if there is any truth in the homeopathic claim of success, it is due to—giving nothing.

